# Intentionality and Presence: On the Intrinsic Of-ness of Consciousness from a Transcendental-Phenomenological Perspective

**DOI:** 10.1007/s10743-012-9106-5

**Published:** 2012-04-27

**Authors:** Wolfgang Fasching

**Affiliations:** Vienna, Austria

## Abstract

This paper discusses the nature of consciousness’ intrinsic intentionality from a transcendental-phenomenological viewpoint. In recent philosophy of mind the essentially intentional character of consciousness has become obscured because the latter is predominantly understood in terms of “qualia” or the “what-it-is-like-ness” of mental states and it is hard to see why such subjective “feels”, of all things, could bestow states with objective reference. As the paper attempts to demonstrate, this is an inadequate understanding of consciousness, which should instead be defined in terms of presence. Consciousness essentially takes place as presence-of, i.e., consists in something coming to appearance. This presence-of is not only a fundamental, irreducible phenomenon, but also in a radical sense un-naturalisable. Naturalism only knows “nature”, as the world of objects, and the question of intentionality then seems to be how certain inner-worldly objects can be “representations” of other inner-worldly objects. In fact, no object is ever intrinsically “about” anything. This is exclusively the nature of *subjectivity* qua consciousness, which is not an object alongside other objects but rather exists as the *manifestation* of objects.

## Introduction

Intentionality is the characteristic of mental states of being *about* or *of* something: to perceive means to perceive something, to think means to think about something, to remember means to remember something, and so forth. This seemingly trivial fact, which we simply take for granted in our everyday lives, proves on closer reflection to be quite hard to comprehend. How does it come about and *what does it mean at all* that something is “about” something, that a particular occurrence “refers to” or “is directed at” something else? For example, what makes it the case that my present thinking is not just a succession of inner-subjective states, but precisely *thinking about* something, i.e., that it in some peculiar way *reaches out* to things transcendent to it?

In recent decades the attempt to “naturalise” the intentionality of mental states has been predominant in philosophy in that regard. Guided by the fundamental materialistic paradigm of contemporary philosophy of mind, the project is to establish, either, eliminativistically, that the mental property of intentionality does not really exist at all, or, reductionistically, that it is reducible to non-mental properties and relations. Eliminativism about intentionality (e.g. Churchland 2002)—being a thesis *about* the world and our place in it that claims that nothing was ever “about” anything—seems to be plainly self-defeating and therefore a nonstarter.^1^ This leaves the reductionist version of the naturalisation project^2^: Intentionality, like everything mental, is viewed as not belonging to the basic, non-reducible features of nature; consequently it must consist in something else, more basic (Fodor 1987, p. 97). And this means that it must (in principle) be possible to formulate, in non-intentional terms, (at least) sufficient conditions for the existence and the specific content of an intentional state—usually in terms of causal relations (e.g. Fodor 1987; Dretske 1981) and/or natural indicator functions (e.g. Millikan 1984; Dretske 1995).

The naturalism that underlies this project is essentially an objectivism: What truly exists is the objective, and therefore the mind too has to be understood in purely objective terms. One of the consequences of this presupposition is that in today’s philosophy of mind (in contrast to Brentano, who originally introduced the term, and the phenomenological tradition based on his thought), intentionality is largely regarded as being logically independent from consciousness and its notorious subjectivity. This is what Horgan and Tienson (2002) dubbed “separatism” and Kriegel (2003) the “independence assumption”. The existence of intentionality is, according to this view, in no way dependent on consciousness, and if it is *conscious* states that are intentional, they are not so by virtue of their conscious (“phenomenal”) qualities. In this way the mental property of intentionality can be kept free of the problems that notoriously afflict the objectivistic reduction of consciousness, and the realm of the subjective can at least be marginalised, restricted to some elusive “phenomenal qualities” that do not further affect the other, more interesting features of the mental (Zahavi 2003b). Or it is even hoped not only that intentionality need not be conceived of in terms of consciousness and its subjectivity, but that consciousness can perhaps ultimately be accounted for in terms of—purely objectivistically/naturalistically conceived—intentionality, be it along the lines of a higher-order representation theory of consciousness (e.g. Rosenthal 2002) or of representationalism (e.g. Dretske 1995; Tye 1995).

In recent years, however, a growing number of authors have been insisting on the existence of a genuine conscious or phenomenal intentionality, i.e., a form of intentionality to which the phenomenal properties are essential and which therefore can*not* be comprehended independently of consciousness (e.g. McGinn 1997; Siewert 1998; Horgan and Tienson 2002; Kriegel 2003; Pitt 2004). Some even maintain that conscious intentionality is the only case of *genuine* (non-derivative) intentionality, and that any other aboutness is either only metaphorically ascribed or dependent on the intentionality of consciousness (e.g. Searle 1992; Strawson 2010).^3^

John Searle, for example, famously argued—with his “Chinese Room” thought experiment—against the computational understanding of the mind, that a non-conscious computer could never possess real intentionality since it merely manipulates symbols purely according to their formal (“syntactical”) properties, without these symbols *meaning* anything (having a “semantical” content) to the computer itself (Searle 1980b). It is only to us that the outputs of the computer have any meaning, which is due to the fact that the computer was built in a way that its outputs can be sensibly *interpreted* as representing something—by us, who *possess* intentionality. Consequently, the computer cannot provide an adequate model of the intentionality of our own mental states: If they would have to first be interpreted as being about something, we would again have to account for the intentionality of the interpreting acts (i.e., for what makes *them* be about the interpreted states), and so on ad infinitum. In order to avoid such an infinite regress, we must somewhere posit what Searle calls “*intrinsic* intentionality” (Searle 1992, pp. 78–82), i.e., something which has intentional content not only for an intentional observer, but in itself. And this is precisely the intentionality of our own subjective experiences, which consequently are fundamentally different from computational states. Computers only possess “as-if intentionality” (ibid.), insofar as they to a certain degree behave *as if* they had intentionality.

Now a proponent of the naturalisation of intentionality would concede that a mere succession of computational states according to formal rules does not in itself bestow these states with representational content (see e.g. Fodor 1982, pp. 284–285); rather, these states also have to be causally connected to the world in the right way. For example, one could hold that if a computer were built into a robot which receives information about its surroundings via sensors and uses this information for the environment-adequate regulation of its behaviour, it would be perfectly justified to say that the information-bearing states “represent” the respective environmental conditions—and not only *to us* as external observers, but *to the robot itself* which takes its internal states *as* bearing information about its surroundings. This “robot reply” (as Searle calls it) corresponds in principle to the basic idea of the program of naturalising intentionality, according to which—broadly speaking—inner states have their intentional content by virtue of their causally co-varying with certain environmental conditions and/or their having, for the actions of the system, the function of indicating such conditions.

Searle’s answer to the robot reply (and therefore implicitly to the whole project of reducing intentionality to causal relations) is basically that the causal relations in which the states of a computer might stand—i.e., to what causes them and to what, in turn, they are triggering (as “behaviour”)—are completely irrelevant to the computational processes in themselves. The computer still only deals with its internal states and their formal properties and has not the slightest idea what these states are “about” (Searle 1980b, p. 420). In order to bestow these internal states with representational content *for* the computer, these causal connections would themselves somehow have to be there for the computer, i.e., it would have to know of them—but then “we are no longer explaining intentionality in terms of symbols and causes but in terms of symbols, causes, and intentionality” (Searle 1980a, p. 454; see also Searle 2002, p. 672).

For Searle, intentionality is irreducible to objective processes; it is rather a feature of (irreducibly subjective) consciousness, which is the only original locus of intrinsic intentionality (Searle 1992, pp. 157–159, 162–164): Only our conscious experiences^4^ need not be interpreted as having intentional content, but simply have it. Without consciousness, nothing is ever really “about” anything.

Now, if we grant a certain plausibility to this intuition about the essential connection between intentionality and consciousness, the question arises as to what is so special about consciousness that it manages to do what is utterly impossible without it. Why should it be the *being*-*subjectively*-*experienced* of states, of all things, that bestows them with *objective reference*?^5^

In light of the currently prevailing understanding of consciousness, this seems obscure. Today, “subjectivity” is understood mainly in the sense of “being only subjectively (privately) accessible”: Conscious experiences seem to have the peculiarity of not being directly observable “from the outside”, i.e., of being evident only to the subject that has them, and this raises all the well-known problems of their reducibility to the—publicly accessible, “objective”—physical. And it is hard to see why the “being only subjectively given” of states should be constitutive of their intentional relatedness. In this paper, in contrast, I comprehend “subjectivity” primarily in terms of the subject-object relation. In this sense, “subjectivity” refers to the *being*-*there*-*for*-*me* of objectivity, i.e., the *consciousness*-side of the consciousness-of-something relation. In other words, subjectivity in this sense does not primarily mean being accessible only from one point of view—being an *object* with the peculiarity of possessing a highly restricted (“idiosyncratic”) accessibility, as Rowlands aptly puts it (Rowlands 2008, pp. 284–285)—but *being* a point of view, the access itself. Consciousness is the *seeing* of objects, and not one of the objects seen (ibid.; see also Rowlands 2010, p. 168).

Accordingly, I will maintain in this paper that the intrinsic intentionality of consciousness is to be understood as disclosure (coming-to-appearance, presence-of-something-to-someone) which is an irreducible (basic) category, and an irreducibly subjective one (i.e., irreducible to any objective relations and therefore uniquely pertaining to consciousness). Appearing-of-reality is a fundamental feature of reality, and one that takes place in, and only in, consciousness. This is the guiding idea of this paper.

To give a short overview of what is to follow: The general intent of this paper is to discuss the nature of the intrinsic intentionality of consciousness from the perspective of transcendental phenomenology.^6^ In Sect. 2, I shall develop an understanding of consciousness (qua taking place of givenness-of) as being essentially intentional. In Sect. 3, I will argue that this intentionality, although it is due exclusively to the intrinsic nature of consciousness itself and therefore exists absolutely independently from what goes on in the “rest of the world”, is nonetheless not merely a matter of what is immanent to consciousness, but one of true world-relatedness. The paradoxicality of this state of affairs leads me to very briefly address—in Sect. 4—the transcendental-idealist view of the consciousness-world relationship, according to which the world is in some sense just as intrinsically consciousness-related as consciousness is world-related.

## Consciousness as Presence-of

That consciousness is intentional means that it is consciousness-*of*. And to be conscious of something means that this something is *there for me*, *appears to me* (in one way or another: as actually present, as past, as possible etc.). It is impossible to further explicate (in terms of something else) what is meant here by “thereness” or “appearing”, since this is an absolutely primitive phenomenon, or rather the very fact of phenomenality itself. One can only point to it and hope that everyone knows what it means that something is present to her or him.^7^

Now, the crucial point is that this of-ness of consciousness is not something that—by virtue of some external relations it might have to other things—is *added* to the being of consciousness, rather consciousness *is* in itself nothing but the taking place of appearing, i.e., it has its being in its of-ness. When I, for example, reflect on my perceptual experience of the glass in front of me, I do not find some “inner occurrence” and then have to additionally ask whether it stands in some representational relation to something else; rather, it only exists as the being-visually-given of the glass from the outset. This is the lesson of the so-called “transparency” of consciousness, i.e., of the fact that when I attempt to observe my consciousness I end up finding the *object* I am conscious of. This is simply due to the fact that consciousness is nothing but the presence of what it is consciousness of—and one cannot find the presence of something without ipso facto finding this something whose presence it is.^8^

Hence intentionality is intrinsic to the being of consciousness. “Intrinsic intentionality” means in this context not only that it is not derived from the intentionality of other states, but that it is not dependent on anything beyond consciousness at all. Intentionality belongs to its being itself; consciousness *consists* in the coming-to-appearance of what it is consciousness of. This is a remarkable and unique mode of being. While a physical state of a system that may function as a representation of this or that (or is interpretable as a representation of this or that) could as well exist as precisely this physical state *without* functioning as a representation (namely if it stood in different relations to other states within and without the cognitive system), it is absolutely impossible that a conscious state could exist as precisely this conscious state, exactly as it is subjectively experienced, without being consciousness of whatever it is consciousness of. A conscious experience is, again, not something in itself that *additionally*, by virtue of its embeddedness in a certain network of causal relations, stands in some representational relation to something else; it is in itself nothing but that taking place of the being-present or givenness of what it is conscious of (see Hua XIX/1, p. 386).

So “intentionality of consciousness” means that consciousness exists as presence-of. This is something completely sui generis: No objective “representational relations” that I may find within the world are even remotely comparable to it. Therefore it misses the point to conceive of intentionality in terms of such relations and then regard the intentionality of consciousness as a *case of* intentionality in this sense. Consciousness qua taking place of presence-of has nothing whatsoever to do with the mapping of something to something, isomorphisms, co-variations, indicator functions, or whatever other objective relations naturalistic theories of intentionality may bring into play: No objective relations between objective entities can ever, taken in themselves, bring about something like the appearing-of-something. Only consciousness can do this (or rather: *is* this). Without consciousness, nothing is present to anyone; and as soon as consciousness exists, presence-of-something already takes place, purely by virtue of the intrinsic essence of consciousness itself and not by virtue of something else that would be external to consciousness.

This is the reason for the (I suppose) quite common intuition that a non-conscious machine could never *really* perceive anything, think about anything, doubt anything, etc., but only, at best, behave *as if* it would do so. Regardless of how elaborate its computations may be, in whatever causal relations they may stand to the environment and how “intelligent” its output may be (interpretable *by us* as representations of the environment), for the computer itself nothing is there at all, since no “thereness” (consciousness) occurs in the first place. Intentionality as we know it from our own conscious states is the phenomenon that consciousness exists as the appearing-of-something. To say that a non-conscious machine could be in the same intentional states as we are, only without consciousness, is therefore simply nonsensical: It amounts to saying that without consciousness, something can appear too, only without appearing. Hence, however adequate it may be to say that a physical state of an information processing machine “is representing something”, and however literally or metaphorically such formulations may be understood, it is not only not sufficient to account for the of-ness of our states of consciousness; it simply has nothing whatsoever to do with it and therefore contributes nothing toward explaining its nature. And it is *this* kind of of-ness for which the term “intentionality” was originally introduced, and which constitutes a central explanandum in philosophy of mind.^9^

This simple and entirely obvious fact has been obscured in recent philosophy of mind by the latter’s conceiving of consciousness primarily in terms of “qualia” or the “what it is (or feels) like” to undergo an experience (i.e., of subjective experiential qualities of some mental states). These qualities are distinguished as the “phenomenal” aspects of the mind from the intentional aspects, as if consciousness were something that only contingently accompanies our cognitive-intentional processes (see Zahavi 2003b). The phrase “what it is like” was originally introduced, properly, to point out the subjective (objectively not sufficiently characterisable) nature of our experiences, yet its current ubiquity has tended to set the whole consciousness-debate on the wrong track: It suggested that the irreducibility of consciousness exclusively pertains to some elusive “feels”, i.e., some qualitative aspects of mental processes which otherwise are completely objective goings-on that can be perfectly well investigated from a third-person viewpoint—something that has to be explained *in addition* to our cognitive performances. From this perspective it remains entirely unclear which special relevance consciousness should have for the intentional character of mental states: Why should some subjective experiential qualities, of all things, be decisive in determining whether states have objective reference?

For phenomenology, however, consciousness is in no way just a matter of some subjective feels. Rather, it is the happening of coming-to-appearance of something—and this is not something that *accompanies* intentional states but precisely what the intentionality of our experiences *consists in*. It is not the case that without consciousness things would be present as well, only that *with* consciousness there is also some feel to it; without consciousness, nothing would be present to anyone at all.^10^

The answer to the question of why consciousness as intrinsically intentional should be the source of all other aboutness is, therefore, simply: Consciousness, and only consciousness, intrinsically exists as presence-of. That appearing-of happens is a primitive, not further analysable fact—precisely the fact of consciousness. Accordingly, the intentionality of our mind shares the irreducibility of phenomenal consciousness, or rather: it *is* phenomenal consciousness, if we understand “phenomenality” in the literal sense of the Greek *phainesthai* (“appearing”, i.e., the “phenomenal manifestation” of something).^11^

One could say: This amounts to answering the question of how consciousness manages to represent something by saying it just does; and one could hold that this is not a very satisfying explanation. Yet—that’s simply the way it is. To be the presence of something is simply the mode of being of a conscious state, and it does not have its of-ness by virtue of anything else. The intentionality of consciousness is an irreducible feature, hence, there *can* be no “explanation” of it, if we mean by this a *reductive* explanation.

### First Specification: Noetic Characteristics

Consciousness as being intrinsically the presentness of what it is conscious of (i.e., as performance of the being-given of its respective object) is the basic intuition of phenomenological thinking. Accordingly, phenomenological reflection is not a turning-away from the objectively given world in order to investigate a realm of subjective goings-on, but an investigation of this very world itself, namely *as* given. The decisive step into the transcendental-phenomenological attitude consists precisely in leaving the reifying apprehension of consciousness as an object within the world of objects (and thereby as a sphere of “subjective interiority” in contrast to the “outer world”) in favour of an understanding of consciousness as the *coming*-*to*-*appearance* of any objectivity. This is the basic idea of the phenomenological reduction which leads to the “transcendental” (in contrast to the “psychological”) consideration of consciousness.

That consciousness has its being in the appearing-of-something does not mean that nothing but the experienced objects and their properties are to be found in it. I can, according to Husserl, investigate the phenomenal both in noematic and in noetic respects. “Noema” is a terminus technicus of Husserl’s for the object-as-it-appears (i.e., considered exclusively with regard to the way it is given in the respective act) (Hua III/1, p. 203)^12^; “noesis” designates the act of consciousness *in which* the noema is given. Now, the realm of the phenomenal can be investigated not just noematically (i.e., with regard to *what* comes to givenness) but also noetically (with regard to *which conscious acts* it comes to givenness in). For example, acts can differ—as Husserl elaborates in *Logical Investigations*—(a) with regard to their “act-quality” (judging, doubting, questioning, wishing, etc.) and (b) with regard to their “act-matter”, the “apprehension-sense” (*Auffassungssinn*): i.e., there are different *modes* of referring to objects, and we can refer to objects in different *respects*. These are characteristics of our conscious acts, and not objective properties of objects. Yet this distinction between noematic and noetic investigation should not be misinterpreted as meaning that we would find a noesis *apart* from the respective noema, as an entity of its own—rather the noetic *is* nothing but the bringing-to-presence of a corresponding noematic aspect. Where should I find the act-quality of “positing” (taking-as-real) other than in the being-posited (being-there-for-me-as-real) of the posited? Where should I find my doubting other than in the being-present-as-doubtful of the doubted (and analogously for all thetic modalities)? And the act-matter manifests itself nowhere other than in what respect, i.e., *as what*, an object is given respectively.^13^ The various kinds of act are nothing other than different ways of the object being there for us (Hua XIX/1, pp. 394–395). Consequently, noetic moments cannot be found apart from the noematic correlate, the given-object-as-given (Hua III/1, pp. 227–228), for they *are* nothing but the presence (consciousness) of the respective noematic moments.

The noema is the object-as-appearing, the noesis is the appearing-of-the-object. These are not separately locatable entities, but only different respects in which one and the same unitary happening of manifestation can be thematised. The difference between the two respects is that the noema forms a unity in contrast to the manifoldness of the noeses manifesting it, i.e., the manifoldness of givenness-events in which the given comes to givenness *as* one and the same (Hua III/1, p. 231). That is, even though with each change on the noetic side there necessarily is a change of some noematic aspect, there is a form of identity we find in the noema—the identity of the same appearing object—which is not to be found in the permanent flux of the noeses. So the difference between noesis and noema is the difference between the single presence-instance and what is present in it yet which can have its presence *as* the same in manifold such single presence-events. Nonetheless noesis and noema are not separately existing entities but only two (distinguishable, but not separable) aspects of one and the same happening of manifestation: We find the object only in its appearing, and the appearing is always and essentially the appearing of what appears.

### Second Specification: The Question of Non-Intentional Consciousness

One could grant that consciousness is always and essentially consciousness-of (i.e., that consciousness consists in some content being present), yet doubt that this, taken by itself, helps to explain intentionality. One could hold that although every consciousness is consciousness of a content “the ‘of’ of ‘conscious of’ is not always the ‘of’ of intentionality”, as Searle says (Searle 1992, p. 84). Intentionality means that something refers to something other than itself. And one could maintain that, e.g., in a mere bodily sensation (say, a pain) doubtless something is experienced (present), but that it is not something distinct from the state of consciousness itself that comes to givenness here; i.e., that appearance takes place—this being the definiens of consciousness—but without this appearance being the appearance of something transcendent to this very appearance: “If I am conscious of a pain, the pain is not intentional, because it does not represent anything beyond itself” (ibid.). And similarly it could be asked with regard to moods (at least to some of them), whether they present us with some *object* or rather just with themselves. I can, for example, be in a state of melancholy without being melancholic *about* something in particular. Consequently, the intentionality of consciousness must consist in something other than just in the undeniable fact that consciousness always has, by virtue of its mere being, a content.

I wish to consider three (not mutually exclusive) replies to this objection against the identification of consciousness and intentionality.

First of all, one could argue that the supposed fact that the consciousness-“of” is not always the “of” of intentionality need not necessarily mean that the essential of-ness of consciousness contributes nothing at all to understanding the intrinsic intentionality of *intentional* states of consciousness. Conscious intentionality is in any case a special *form* of the appearing (presence-of), which constitutes the essence of consciousness. Even if not all appearance is the appearance of something transcendent to this appearance (and therefore intentionality), it nonetheless remains the case that conscious intentionality is a case of appearing and can only be understood in terms of the essence of consciousness qua taking-place-of-presence, and not independently of consciousness (as the separability thesis has it). Conscious intentionality is a case of the of-ness of consciousness—namely the case in which something is given as in its being not being exhausted by this givenness—and not something that is externally added to the consciousness-“of”. If no appearing takes place, nothing can appear as being transcendent to this appearing (i.e., as an object).

Nevertheless, there seems to be a gap between the mere consciousness-of-ness and the intentional of-ness, and one could therefore doubt that the former already renders the latter intelligible. Now one could, secondly, deny that there is any non-intentional consciousness at all. One could hold that the so-called non-intentional conscious experiences are not really concrete experiences but rather dependent, only abstractively distinguishable, *moments* of the happening of the intentional coming-to-appearance-of-transcendence which constitutes the being of consciousness (Hua III/1, p. 191). Something like sensations only exists in the medium of intentionality: Sensation is really nothing other than sensing, and that is the being-sensuously-there of a perceived object. A tactile sensation, for example, *is* nothing other than the tactile presence of the touched surface (and simultaneously, if in a fundamentally different way, the presence of one’s own touching body); and a pain which, in contrast to the tactile sensation, may in itself not be the givenness of an “external” object, is nevertheless a mode of my body’s being present to me, as well (see e.g. Crane 2003). As for moods, they are likewise not something we would find independently of the givenness of the world in some subjective “inwardness”, but rather are—as especially Heidegger has elaborated—nothing but precisely aspects of the way the world is there for me (see e.g. Seager 1999, p. 47; Crane 2003; Zahavi 2003b, p. 72). Where, if not in the way things appear to me, should I experience my melancholy?

According to this view, the phenomenologically fundamental is the concretum of world- and object-appearing (the *Erscheinen*-*des*-*Erscheinenden*) upon which I can abstractively distinguish certain moments which, however, in no way possess any independent existence. Just like the aforementioned thetic and aspectual moments, the hyletic moment, too, is something that I only find in the given object (qua given). Just as I do not find the “act” apart from the appearing object but only as its respective mode of being there for me, the hyletic, too, is not an independently existing sense-data material whose being-apprehended as manifestation of an object would be extrinsic; it is nothing other than the sensuous mode of presence of an object. Both the act and the hyletic components of consciousness are encompassed by the total phenomenon of appearing-of-something, i.e., are only dependent moments of intentional experience.

Now one could say that even if it holds that sensations are only dependent moments of intentional consciousness, they are nonetheless something we are conscious of, and consequently we find *within* intentional consciousness an of-ness that is not the “of” of intentionality. To this, it can be replied, thirdly—and this is the most fundamental of the three replies, since it calls into question the strict distinction between intentional and non-intentional of-ness in general—that even my pain as such is, in an ultimate sense, not “part” of my consciousness, but precisely something I am conscious *of* and which already is a kind of transcendence. Thus consciousness-of-pain is intentionality, and consciousness is intentional through and through. Even the original givenness of subjectivity-“immanent” contents displays a certain appearance-transcendence from the start, which manifests itself primarily with regard to the *temporal* aspect of their givenness (Hua XVII, pp. 290–292). Immanent contents are always given temporally, as streaming; and a non-temporal, non-streaming givenness of such contents is not even conceivable. This implies, as Husserl has shown in his analyses of time-consciousness, that they are given in continuously changing temporal modes (“temporal adumbrations”). They are given as coming and going, so that one and the same temporal phase is given *as* the same in continuously changing modes of givenness—as present (in “primal impression”), as sinking back (in “retention”), as sinking back more and more, etc. This constitutes something like a primary form of objectivity, a proto-objectivity so to speak, by something being given as the same in changing consciousness and therefore as existentially transcending the respective appearance, even if not appearance as a whole. Accordingly, already in the givenness of sensations we are dealing with a givenness-as-transcending-this-givenness. A sensation is consciousness-“immanent” in the sense that, in contrast to “external” objects, it has its *being* in its givenness; yet already within this (existence-constitutive) givenness a play of transcendences in which one givenness refers to other givenness unfolds, the given being given as also being given in other givennesses. We are dealing here with a primal transcendence (primal objectivity) within immanence (subjectivity).

Hence we can distinguish different levels of transcendence. A pain could not exist without being given as temporally extended and streaming in each of its phases, and it is in this sense “immanent” to its givenness—nonetheless this immanence already implies something like primordial transcendences, precisely because of the essential temporality of the givenness. Immanent data possess an even higher degree of transcendence with regard to re-enacting *memories*, since the givenness-in-memory is, in contrast to retention, not constitutive of the being-given-at-all (and therefore the being) of the remembered experiential contents (so that memories can be mistaken). Accordingly, memory constitutes a first objectivity proper of one’s experiential stream (Hua XVII, pp. 165–166, 291). Still higher forms of experience-transcendence display the givenness of other subjectivities and the givenness of external objects, the latter of which are given as existing independently of *any* givenness.

So the Husserlian analyses unearth something like “micro-intentionalities” that function *within* intentionality in the usual sense. In this view, transcendence is given in immanence, yet this immanence is itself a transcendence with regard to a more radical immanence. Consciousness itself, in which all levels of transcendence come to givenness—the true and ultimate immanence, which Husserl calls “absolute consciousness”—does not “consist” of some immanent contents or data at all, but is nothing other than precisely consciousness *of* contents, pure intentionality. “Consciousness of a sound is not itself a sound and does not contain it as a part. A sound is not a consciousness” (Hua XXXIII, p. 160). In this sense, for phenomenology there *is* ultimately no non-intentional sphere in consciousness, and to say that consciousness is essentially consciousness-of is already to say that it has its being in intentionality.^14^

In any case, even if one does not grant this point and insists that somewhere in consciousness there must exist a purely “immanent” and therefore non-intentional givenness (which, in light of the radicalised conception of intentionality of point (3), can only be, in the sense of point (2), a dependent *moment* of intentional consciousness), there nevertheless remains the argument of point (1) that intrinsic of-ness is a *case of* the presence as which consciousness essentially exists. And this suffices for our present purpose.

## The Indubitability of Conscious World-Relatedness

A natural objection might seem to be that nonetheless the notion of consciousness as presence-of does not really help us understand the nature of intentionality, after all: One might concede that consciousness essentially consists in the presence of some content, yet argue that precisely the fact that this is an essential, intrinsic feature of consciousness, which exists utterly independently of what goes on in the rest of the world, shows that what is present in this sense are only *subjective*, *inner*-*mental* objects whose relation to the outer world remains to be investigated. Hence the definition of consciousness as presence-of has not got us any closer to an understanding of intentionality. The question was, after all, how something inner-mental can represent something external, and it is unclear why this should be any more obvious with regard to subjective-phenomenal contents than with regard to physical states of a cognitive system. In other words: That there are “phenomenal appearances” in consciousness is all well and good—but the real question of intentionality is how the mind manages to refer via these appearances to something outside consciousness, i.e., what accounts for the representational relation of these subjectively given contents to something external. And this cannot be exclusively a matter of what goes on *within* the mind. Thus, if conscious states are intentionally related to something, they cannot be so merely by virtue of their conscious or “phenomenal” qualities.^15^ This is precisely the point where the causal or other naturalistic theories of intentionality are applied in an attempt to elucidate what kinds of natural relation between system-states and the environment of the system make these states into representations-of-something—where the question of whether these states are subjectively experienced (or present) plays no relevant role.

Yet actually, the phenomenological determination of consciousness as presence-of is radically misunderstood when it is characterised as the existence of subjective (merely inner-mental) contents. The problem with this characterisation is the simple and fundamental fact that the intentional relation itself to the outer object is a conscious one (and not something that is somehow externally added to the conscious state by objectively existing relations between the state and other things). It is *not* just an inner-subjective content that is consciously there for us (perhaps “standing for” something external, which, however, cannot be decided exclusively on the basis of what is immanent to consciousness^16^), but the object itself. And as Husserl has convincingly argued, the being-consciously-there-for-us of an object cannot be accounted for by appealing to the mere existence of inner pictures or other representations, in whatever relations of causality (see e.g. Hua XXXVI, p. 58) or of isomorphy (see e.g. Hua XIX/1, pp. 436–440; Hua XXXVI, pp. 106–107) or whatever other external relations they might stand to other things. In *Logical Investigations* he argues against the “picture theory”, i.e., the idea that outer objects are given to us by something like pictures of them existing in our mind: “What […] enables us to go beyond the ‘picture’ which alone is present in consciousness and to relate it *as* a picture to a certain extra-conscious object? To point to the resemblance between picture and thing will not help. It is, at least if the thing really exists, doubtless the case as an objective fact. *Yet to consciousness, which ex hypothesi has only the picture, this fact is simply nothing at all*; it therefore cannot serve to explain the nature of the presentational […] relation to the external object” (Hua XIX/1, p. 436; 2001, p. 125: translation adapted, italics partly mine). A picture only functions as picture for us (and the same holds, as Husserl stresses, mutatis mutandis for any kind of representation: ibid., p. 438) by its being apprehended *as* picture-of, i.e., only because *in* the picture something else (the pictured) is there *for* consciousness—hence only because consciousness intentionally reaches out through the presence of the picture to the outer object and *not* only the picture is present to it. Thus, the existence of contents which function as representations-of to consciousness presupposes consciousness’ being-intentionally-related-to-the-represented and cannot explain it.^17^ So one cannot say that what is present in consciousness are only immanent data after all, and that they are only interpreted by us as representing an outer world, for such an interpreting-as *is* precisely a form of the being-present-to-us in question.

So the point is that the mere existence of an alleged mental representation (in the sense of an immanent content that stands in some objective correspondence-relation to something else) cannot account for the intentional being-related-to-something of consciousness. For by “intentionality of consciousness” we do not just mean a supposed objectively existing representational relation in which the subjective consciousness might stand to the outer world, while being *to* this consciousness nothing at all; we mean *conscious* intentionality, i.e., that one is *consciously* related to reality itself (intentionality as being itself an experiential fact, and not only an external relation between experience and its outside). It is the object itself that is present to us, and “a transcendent object is not present to consciousness merely because a ‘content’ rather similar to it simply somehow *is* in consciousness […] but because all relation to its object is part and parcel of the *phenomenological essence* of consciousness, and can in principle be found only there, *as* a relation to a ‘transcendent’ thing” (Hua XIX/1, p. 437; 2001, p. 126: translation adapted).

If we do not presuppose the world-presencing character of consciousness as belonging to its intrinsic essence (i.e., if we take consciousness as in itself being nothing but an agglomeration of immanent experiential data), it remains entirely incomprehensible how we could ever *leave* this immanence—how we could, for example, ever consciously think about the relation between our consciousness and the outer world and develop causal theories of intentionality (see e.g. Hua VII, pp. 119–120). All scientific insights that might inform us about the relation between our subjective interiority and the objective world would then be, conscious-wise, merely a further agglomeration of subjective contents, which might objectively stand in ever more elaborate correspondence-relations to the outer reality, yet without consciousness itself ever knowing anything about this because there would not be any conscious knowing in the first place. No scientific effort could ever establish a conscious relation to the world, i.e., make it be something *to* consciousness itself. Thus the naturalistic theories of intentionality, which conceive of it as consisting in external, objective relations between internal states of the subject and the external world, leave completely unintelligible the very being-there-for-us of objective reality they necessarily permanently presuppose and draw upon.

One cannot appeal to inferences (e.g., “inferences to the best explanation”, see Fodor 1982, p. 293) as an explanation of how we transcend the subjective data we are primarily confronted with toward an external reality. We could not even ask about the true character of reality, let alone infer to it, if this reality were not in principle already there for us, even if we may be in the dark about its true nature. The opened-up-ness of reality in consciousness is presupposed by any inference and therefore cannot be *established* by inferences. Any inferring from something to something takes place within the medium of the thereness-for-us of these “somethings” as which consciousness exists from the start (Hua XXXVI, pp. 39–40, 174–179).

Of course it is possible that science informs us that the way reality pre-scientifically appears to us is “only subjective” and that its true nature is actually quite different. This means nothing more than that reality now, in light of scientific investigation, appears as being different from how it first appeared. Yet this only makes sense if it was reality itself that appeared in the first place. Science might inform us that reality is *different* from how it first appeared, but it does not thereby refer to *another* reality besides the “subjectively appearing” one: it refers precisely to the reality that is there for us from the start, only further determining its nature according to specific methodological ideals (Hua III/1, pp. 110–116; Hua VI, p. 50). Even in the most “subjective” sensuous perception, the subject is not enclosed in the interiority of its subjectivity but exists as the disclosure of transcendent objects (Hua XXXVI, pp. 90–91); and, on the other hand, in no science, however “objective” it may be, do we ever deal with something other than the subjectively appearing world (Hua III/1, p. 113).

What I have attempted to say in this section is that if it is not reality itself that is present to us by virtue of the intrinsic essence of consciousness, we cannot even *ask* about the alleged causes of the “internal contents” and no inference will ever lead us to reality itself. Hence, either consciousness exists as the in-principle disclosedness of the “outside” from the start, or it will *never* reach it. This latter option, however, is a thought *that simply cannot be thought*, because already the thought that we do *not* intentionally reach the real world is about the real world itself (*as* never reached by us) and accordingly presupposes the intentional being-with-the-world it explicitly denies. The intentional world-relatedness of consciousness is therefore an absolutely indubitable fact that even any doubt must presuppose.^18^

## Prospectus: Alongsideness and Presence-of-ness. The Transcendentality of Consciousness

So the intentionality of consciousness does not consist in any objectively existing, consciousness-extrinsic relations between the “inside” of consciousness and the external world, but is (as I attempted to show in Sect. 2) a matter of the intrinsic essence of consciousness itself. Nonetheless, it is (as I attempted to show in Sect. 3) undeniably a matter of true world-relatedness and does *not* only pertain to the “inside” of consciousness in contrast to the rest of the world. Herein lies something deeply enigmatic that can hardly be comprehended in naturalistic terms (see McGinn 1997, pp. 303–304).

I understand “naturalism” here not only—as is usual today—in the narrow sense as physicalism (i.e., as the thesis that ultimately only nature in the sense of the subject-matter of physics really exists), but in a broader sense as the thesis that everything really existent is part of nature in the sense of the *world of objects*, spread out before us in objective space and time. This does not exclude a “dualistic” view of mind and matter, as long as it integrates the psychical (whose irreducible existence in addition to the physical it asserts), as being just a further “natural phenomenon”, into the objective world and conceives of the relation between consciousness and the rest of the world as analogous to the relations between any other inner-worldly things. In this sense, most of the present anti-physicalistic theories of consciousness are naturalistic, and an account of intentionality could very well accept the irreducibility of phenomenal consciousness and nevertheless conceive of its intentional world-relatedness naturalistically, namely in terms of natural relations between inner-objective occurrences.

Hence naturalism in this sense is tantamount to objectivism, which only knows of the world of objects and cannot but conceive of conscious experiences as merely being further objective occurrences, existing side by side with the other inner-worldly occurrences. This is the form of naturalism Husserl was primarily confronted with and his staunch anti-naturalism was opposed to (see, e.g., Hua XXV, pp. 8–9, 13; Hua VI, p. 224), claiming that it results in a “blindness to the intentional” (Hua VII, p. 122; and ibid., p. 106) since it can understand conscious experiences only as—for some awkward reason only “subjectively accessible”—objects among objects, instead of the *access itself* to the objective. In other words, by knowing only of the world of what is objectively given to us, objectivism makes the very givenness itself of the objects which takes place in consciousness unintelligible.

Naturalism cannot but understand our consciousness *of* things as a further thing. The problem then is: How can a thing intrinsically be the presence of another thing? One thing can bring something else to presence by being there *for* consciousness *as* representation of something else, yet obviously this does not help us understand the being-there-for-consciousness itself. *In itself* no thing is ever the thereness of something else. A thing simply is what it is, and any relatedness it might have to other things can never be purely a matter of what is intrinsic to this thing alone. Thus, if a conscious experience is, as naturalism would have it, only a natural occurrence that exists *side by side* with other natural occurrences (maybe standing in some correspondence-relations to other things and causing environment-adequate behaviour),^19^ it remains totally incomprehensible how our experience in itself can have any significance with regard to what exists outside of it.

This is precisely what made intentionality appear so enigmatic to Husserl. In his view, it is impossible to comprehend intentionality as long as we conceive of the consciousness of an object as a “second object” (Hua XXXVI, p. 38), existing side by side with the object it is conscious of. In this sense he asks: “Why should successions of perceptions and of cognitive acts based on them, immanent goings-on in consciousness, have any bearing on an *outside* of consciousness […]?”, in order to continue: “Or does it ultimately make no real sense to speak of an ‘outside’ of consciousness” (ibid., p. 40)? Is consciousness perhaps not a second object alongside the object it is conscious of, but “[i]s ‘objectivity’ rather something that belongs to the essence of consciousness so that consciousness is not itself an objectivity but a necessary correlate of any possible objectivity” (ibid., p. 41)? I.e., the object consciousness refers to is, in Husserl’s view, not an outside of it simpliciter (just as one thing exists outside of the other), but precisely the “outside” as whose thereness it essentially exists and which, in this sense, belongs to its own being (Hua XVII, p. 257; Meixner 2006, pp. 37–39). Anything we can ever mean by reality is precisely the reality that is there for us by virtue of the intrinsic essence of our consciousness, i.e., the consciously *appearing* reality.

Of course, not everything that appears also exists. But *if* an appearing thing exists, it is precisely this very appearing thing itself that exists—and not *another* thing (the “real” thing *beyond* the realm of appearance).^20^ So, reality, according to transcendental phenomenology, cannot be something utterly distinct from the appearing world, and the difference between “mere appearance” and “real existence” cannot be one between the realm of appearance and an outside tout court of appearance; it is rather one that constitutes itself *within* the realm of the phenomenal (Zahavi 2003a, pp. 53, 56).

For phenomenology, the world has to be thought in terms of its manifestation, and accordingly, what it means for a thing to exist cannot be understood independently of what it means for it to appear-as-existing: To exist *means* precisely to be, in the intra- und intersubjective process of world-experiencing, consistently findable *as* really-existing within the phenomenally given world as whose thereness our subjectivity essentially exists. Being has meaning only with reference to its appearance and therefore to subjectivity, independently from subjectivity it has no meaning at all (see Hua XVII, p. 279).

Yet then our consciousness—qua the taking place of appearing—is not simply one of the objects, existing side by side with the other objects, but somehow belongs to the very meaning of objectivity itself (Hua III/1, pp. 100–101). So obviously the thing-related category of “outside-of-each-other” cannot be sensibly applied to the relation between consciousness and “other things” (see Gallagher and Zahavi 2008, pp. 124–126). Consciousness exists as the thereness of the realm of objectivity *within which* things can exist outside of each other, whereas the relation between the thereness of things to these very things is not itself to be understood as an outside-of-each-other (Hua XXXVI, pp. 29–30; see also Zahavi 2008).

Hence, Husserl’s claim that “*[t]here is no thinkable point where the life of consciousness is broken through, or could be broken through*, and where we might reach a transcendence that possibly had any sense other than that of an intentional unity having its appearance in the subjectivity of consciousness” (Hua XVII, p. 242; 1978, p. 236: translation adapted) in no way amounts to a “subjectivism” in the usual sense, i.e., the thesis that we never get beyond the immanence of our subjectivity and that anything we ever deal with is “*merely* subjective”. From a Husserlian perspective, such a thesis is nonsensical since it implicitly (by considering the subjective to be “merely subjective”, i.e., in contrast to the rest of the world) draws upon the being-there-for-us of the outside of subjectivity it explicitly denies. Saying that one never gets beyond subjective interiority already presupposes the reaching out to the outside (to which one refers *as* never reached). The idea of consciousness never leaving its immanence only makes sense when it is understood that this does not exclude any transcendence we can even *wish* to reach, and that with this *transcendental* “subjectivism” we have therefore lost nothing at all of the world. In other words: The thesis that the sphere of consciousness is never broken through is nonsensical as long as it is supposed to mean that we never reach the objective world, i.e., as long as the purportedly non-transcendable subjectivity is conceived of as possessing an outside. Rather, we must *radically* embrace the “interior standpoint”, whereby its opposition to objectivism dissolves (Hua IX, p. 300).

I will not pursue this idea any further here, which no doubt raises many questions and would require a thorough discussion of its own. It is clear that not many of us will be willing to accept this “idealist” dissolution of the “paradox of subjectivity”, i.e., the puzzle of how a *part* of the objective world can simultaneously be in itself the *thereness* of this world (Hua VI, p. 182). Then, however, one has to at least admit that there *is* a puzzle, and that there is something deeply un-comprehended in the intrinsicality of conscious intentionality. Naturalism, in any case, is—I am convinced—not a way to a possible solution but rather the inability to see the problem in the first place.

## Footnotes

1 Of course, there have been attempts to answer this charge, and one would have to discuss the matter in more detail to convincingly refute intentionality-eliminativism—which, for lack of space, I will not do here. In any case, I think that Fodor is quite right when he points out that if intentionality should turn out to not really exist this would be the “greatest intellectual catastrophe in the history of our species” (Fodor 1987, p. xii). Yet he is, to an astonishing degree, mistaken about the range of this catastrophe when he continues: “[I]f we’re that wrong about the mind, then that’s the wrongest we’ve been about anything” (ibid.). This misses the point: The catastrophe would not consist in our being radically mistaken about (!) ourselves, but in the fact that we would *not even be mistaken*. Without intentionality, our world-relatedness as a whole would collapse, and with it the world-relatedness of any science (which is supposed to inform us about the objective non-existence of intentionality).

2 Instrumentalism (as espoused by Dennett), for which the intentional vocabulary is only a, with regard to some systems predictively useful, mode of description, could be placed somewhere in between. Insofar as intentional categories are, on this view, more or less nothing but useful fictions, to which nothing corresponds in reality (apart from the “working” of the intentional description for certain systems), it can, for our purposes, be considered as a more tolerant version of eliminativism (or at least non-realism).

3 For an overview of this debate see Montague (2010).

4 And, according to Searle’s “connection principle”, *potentially* conscious states (Searle 1992, pp. 155–162). I do not agree with Searle on this point, however.

5 Of course Searle’s Chinese Room argument, and also the general thesis of consciousness being the exclusive locus of intrinsic intentionality, have been heavily contested by many authors, but this is not a debate I will pursue further here. In this paper, I am not explicitly arguing for the claim that consciousness is the only form of non-derivative intentionality (although I think it is), but only for the claim that conscious intentionality is at any rate something quite unique that cannot be understood in terms of other relations—whether rightfully called “intrinsically intentional” or not. And it is this unique feature that—*if* the Searlean claim is true—is responsible for the fact that consciousness “manages to do what is utterly impossible without it”.

6 In this attempt, I am especially indebted to the work of Dan Zahavi (e.g. Zahavi 2003b, 2008, 2009). Advocacy of the transcendental-phenomenological view of intentionality can also be found in Meixner (2003, 2006). For an extensive discussion of the relationship between analytic and phenomenological theories of intentionality see Szanto (forthcoming).

7 This does not mean that intentional conscious states cannot be analysed. The givenness of something is usually complexly structured, and detailed analyses of these structures can be given: e.g., which interplay of modes of givenness are necessary for the givenness of an object of a particular type, how givennesses refer to each other, how one givenness “genetically” presupposes other, earlier givennesses on which the new givenness builds, and so on. Such analyses are precisely what phenomenology does. Yet the point is that these are analyses of *internal* structures of givenness and presuppose that givenness takes place at all. So givenness (presence of something) *can* be analysed, but *not* in terms of something else.

8 This view might seem akin to “representationalist” theories of consciousness (e.g. Dretske and Tye). Yet actually the main thrust is quite opposed to representationalism in its typical form: While the representationalists usually claim that consciousness can be explained in terms of—naturalistically reducible—intentionality, the view presented here purports that intentionality can, on the contrary, only be understood in terms of—naturalistically irreducible—consciousness. Representationalism argues that conscious experience has no “intrinsic qualities” and that the so-called qualia are actually properties experienced *objects* are represented as having, and consequently something publicly observable and not something “private”—therefore posing no obstacle to a materialistic treatment of consciousness (see e.g. Dretske 1995, p. 65). This line of argumentation seems obscure to me, however: I completely fail to understand how it should follow from the purported fact that everything that is experientially present in consciousness are objects and their properties that the presence itself of the objects is “naturalisable”, i.e., reducible to objective, physical facts. This is simply a non sequitur. The problem of consciousness is not so much the alleged existence of only subjectively given qualities which seem to be irreducible to physical, objectively accessible features, but that anything is given at all, be it “only privately” or “publicly” accessible (see Zahavi 2003b, pp. 76–77).

9 As to the alleged unconscious intentional states we ourselves are supposed to have, I suppose they can be analysed partly (namely when it comes to “sub-personal processes” in our brain) in terms of Searlean as-if intentionality, and partly (when it comes to unconscious beliefs and the like) as dispositions to conscious intentional states. And as Galen Strawson has vividly explained, a disposition is not something intentionally contentful, but precisely a *disposition* to enter an intentional state (Strawson 2004, pp. 287–288). “To think that a disposition is, metaphysically, the kind of entity that can be contentful in itself is a bit like thinking […] that if an object has a fragile disposition, then it already in some sense contains or involves actual breaking” (ibid., p. 288).

10 Kriegel (2003) supports the claim that non-derivative intentionality is dependent on consciousness, namely (following McGinn 1997) for the reason that only in conscious intentionality is something present to a subject. Yet he suggests interpreting this conscious intentionality (along the lines of his “self-representationalist” account of consciousness) as an intentional state (whose intentionality simply consists in certain natural relations) accompanied by a Brentanian “inner awareness”, i.e., as being simultaneously intentionally directed at itself. This inner awareness is, in contrast to “normal” intentionality, not naturalistically explicable at the moment, but may be so, according to Kriegel, some day. This interpretation of what conscious intentionality consists in does not seem very plausible to me, however. It is indeed—and here I agree with Kriegel—the phenomenon of something being present to a subject, but I doubt that this is adequately understood as “naturalistic” intentionality plus inner awareness: (1) to be able to explain anything, the self-directed intentionality would again have to be the presence of the state to the subject; (2) yet even if we admit such a presence as an explanans, the presence of a state which stands in these and those causal relations to this or that (thereby being in a naturalistic sense “intentionally directed” at this “this or that”) is in no way in itself the presence of that to which it has these relations. For this, it would somehow have to be the presence of these very relations, and thereby ultimately of the things the state is related to, which was the explanandum in the first place.

11 For a similar view see McGinn (2008).

12 Here, I follow the so-called “East Coast interpretation” of the noema (e.g. Drummond, Sokolowski, Zahavi) according to which it is not—as for the “West Coast interpretation” (e.g. Føllesdal, McIntyre, Dreyfus)—a Fregean abstract “sense”-entity, *by means of which* an act refers to its object, but the very object itself, taken with respect to its being-given. Extensive argumentation for this view is found in Drummond (1990).

13 Indeed, the act-matter plays no role in Husserl’s later works and becomes completely absorbed by the correlative noematic aspect, namely the “noematic core” as the sense in which an object is given, in contrast to the “full noema”, the given *as* posited, doubted etc., i.e., including the noematic thetic characteristics, the noematic correlates of the act-qualities (see Hua III/1, pp. 298, 305).

14 The self-luminosity of consciousness, which many authors, myself included, regard as constitutive of consciousness—i.e., the idea of presence being its *own* presence—is a topic of its own which I wish to leave out of consideration in the present paper. Here, we are indeed dealing with a radically non-intentional mode of givenness, yet on the other hand this does not so much pertain to one of the contents we are conscious of (as the neo-Brentanian self-representationalists would have it), but rather to the mode of being of consciousness-of itself. One cannot sensibly speak in this regard of non-intentional conscious experiences or even of a distinguishable non-intentional region within consciousness.

15 See e.g. Millikan (1984, pp. 91–92): “[E]very kind of *awareness of* […] is in part an external relation, the inside of the awareness—that feeling part—giving no absolute guarantee that it *is* the inside of a genuine *awareness of* relation.”

16 Fodor, for instance, states that the difference between a computer whose states are not *really* about anything but can only be *interpreted* as meaning something by their users, and our subjective states which *are* representations of something, lies in the way we are causally connected with the world (Fodor 1982, pp. 284–285). Note that this does not make any difference to the cognitive processes in themselves, which are entirely “syntax-driven” (Fodor 1987, p. 20). Hence the logical consequence of this view is that what Fodor says about the computer—“The machine has no access to that interpretation, and its computations are in no way affected by it. The machine doesn’t know what it’s talking about, and it doesn’t care” (Fodor 1982, p. 285)—must hold true for us too: we too, that is, “have no access to the interpretation” of our thoughts and “do not know what we are talking about”, for this is entirely a matter of the external-causal relations between the mental processes and the world, and not something that could be intrinsic to the mental processes themselves.

17 This argumentation is comparable to Searle’s answer to the “robot reply” (see above): The mere objective fact of causal relations cannot explain intentionality as long as there is no knowledge of these relations, which would presuppose what is to be explained.

18 Naturalistic accounts of intentionality, on the contrary, imply that it is thinkable that all of my conscious states actually do not possess any intentionality—namely if my inner-conscious goings-on should *not* stand in the necessary natural relations to the outer world—in which case I actually never meant anything about anything at all and even now, in spite of all appearances to the contrary, am not *really* thinking about the possibility of not being intentionally related to the world (so that this very consideration presupposes what it purports to call into question). See, e.g., Millikan’s scenario of a perfect double of myself who comes into existence by a freak accident, thereby lacking the—according to her bio-teleological theory, evolutionary—relations to the world necessary for constituting aboutness. What, in my view, should rather be taken as a reductio ad absurdum of her naturalistic-externalist account of intentionality, she presents as an illustration of it: “Though possibly that being would be and even would have to be in a state of consciousness exactly like yours, that being would have no ideas, no beliefs, no intentions, no aspirations, no fears, and no hopes. […] This because the evolutionary history of the being would be wrong. For only in virtue of one’s evolutionary history do one’s intentional mental states have proper functions, hence does one mean or intend at all, let alone mean anything determinate” (Millikan 1984, p. 93). For a critique of this kind of externalism, see Seager (1999).

19 To repeat the core idea of Sect. 3: It may suggest itself that, if the inner-mental processes systematically correspond to the external world and are “used” by the organism for environment-adequate behaviour and in this sense serve *for* the organism as representations of the environment, it is very well the case that in consciousness the environment is there for the organism. Yet the problem is that all these are external relations in which consciousness might stand, but which do not change anything about the intrinsic being of the conscious experiences themselves. That the experiences stand in causal dependency-relations to something else and that they, in turn, cause a certain behaviour (environment-adequate or not), are without exception extra-conscious facts that, by themselves, in no way make the environment *consciously* present. All of this can only be relevant to the intrinsic being of consciousness if it is given *to consciousness itself*—i.e., if we are *conscious* *of* our acting-in-an-environment—which presupposes what is to be explained. Accordingly, all talk about the “embodiedness” and “embeddedness” of cognition contributes nothing to an understanding of our conscious world-relatedness as long as embodiedness and embeddedness are not conceived of as *phenomenal* facts, i.e., as *given to consciousness*—namely as pertaining to the phenomenological observation that we are only conscious of a world by being simultaneously conscious of ourselves as being bodily situated in this very world—which *presupposes* consciousness-of as such.

20 And if the appearing thing does *not* exist, it is precisely this appearing thing itself that does not exist. The presence of a non-existent object is not the presence of an “immanent” object that *does* exist yet has no correspondence in the external world, but precisely the presence of an *external* object that does *not* exist (Zahavi 2003a, pp. 19–20).
